# Contrasting plant ecological benefits endowed by naturally occurring *EPSPS* resistance mutations under glyphosate selection

**DOI:** 10.1111/eva.13230

**Published:** 2021-03-29

**Authors:** Martin M. Vila‐Aiub, Heping Han, Qin Yu, Federico García, Stephen B. Powles

**Affiliations:** ^1^ Australian Herbicide Resistance Initiative (AHRI) ‐ School of Agriculture & Environment University of Western Australia (UWA) Crawley Western Australia Australia; ^2^ IFEVA ‐ CONICET – Faculty of Agronomy Department of Ecology University of Buenos Aires (UBA) Buenos Aires Argentina

**Keywords:** EPSPS mutation, glyphosate, relative fitness, resistance benefit

## Abstract

Concurrent natural evolution of glyphosate resistance single‐ and double‐point EPSPS mutations in weed species provides an opportunity for the estimation of resistance fitness benefits and prediction of equilibrium resistance frequencies in environments under glyphosate selection. Assessment of glyphosate resistance benefit was conducted for the most commonly identified single Pro‐106‐Ser and less‐frequent double TIPS mutations in the *EPSPS* gene evolved in the global damaging weed *Eleusine indica*. Under glyphosate selection at the field dose, plants with the single Pro‐106‐Ser mutation at homozygous state (P106S‐rr) showed reduced survival and compromised vegetative growth and fecundity compared with TIPS plants. Whereas both homozygous (TIPS‐RR) and compound heterozygous (TIPS‐Rr) plants with the double TIPS resistance mutation displayed similar survival rates when exposed to glyphosate, a significantly higher fecundity in the currency of seed number was observed in TIPS‐Rr than TIPS‐RR plants. The highest plant fitness benefit was associated with the heterozygous TIPS‐Rr mutation, whereas plants with the homozygous Pro‐106‐Ser and TIPS mutations exhibited, respectively, 31% and 39% of the fitness benefit revealed by the TIPS‐Rr plants. Populations are predicted to reach stable allelic and genotypic frequencies after 20 years of glyphosate selection at which the WT allele is lost and the stable genotypic polymorphism is comprised by 2% of heterozygous TIPS‐Rr, 52% of homozygous TIPS‐RR and 46% of homozygous P106S‐rr. The high inbreeding nature of *E*.* indica* is responsible for the expected frequency decrease in the fittest TIPS‐Rr in favour of the homozygous TIPS‐RR and P106S‐rr. Mutated alleles associated with the glyphosate resistance EPSPS single EPSPS Pro‐106‐Ser and double TIPS mutations confer contrasting fitness benefits to *E*.* indica* under glyphosate treatment and therefore are expected to exhibit contrasting evolution rates in cropping systems under recurrent glyphosate selection.

## 1 INTRODUCTION

From an evolutionary perspective, recurrent use of herbicides on large plant populations is a strong selection pressure for the enrichment of herbicide resistance mutations (Beckie & Tardif, 2012; Gressel & Levy, 2006; Jasieniuk et al., 1996; Maxwell & Mortimer, 1994; Powles & Yu, 2010). The ability of herbicide resistance mutations to spread and increase in frequency is a function of the survival and reproductive success of plants harbouring those mutations under herbicide selection (Cousens & Mortimer, 1995; Neve et al., 2014). As a result, the spread of particular resistance mutations in environments under herbicide selection will depend on the relative fitness (W) of resistant (R) and susceptible (S) genotypes (i.e. the so‐called resistance benefit) (Simms & Rausher, 1987). A herbicide resistance benefit is a measure of the efficiency of a resistance trait in protecting plants from the lethal effect of a particular herbicide dose in a particular environment. Empirical estimations of resistance benefits associated with herbicide resistance mutations are lacking in the literature where efforts have mostly focussed on assessing survival but not reproductive traits (but see Beckie & Morrison, 1993a, 1993b; Sou Sheng et al., 2016; Yanniccari & Gigón, 2020).

Insights into the adaptive resistance evolution to the very widely used herbicide glyphosate are key to predict weed infestations in extensive cropping areas (Baucom, 2019; Gaines et al., 2019). This understanding has benefited from knowledge of molecular and biochemical resistance mechanisms conferred by resistance‐endowing target site point mutations and their associated fitness costs (Funke et al., 2009; Gaines et al., 2019; Han et al., 2017; Li et al., 2018; Sammons & Gaines, 2014; Sammons et al., 2018; Vila‐Aiub et al., 2019; Yu et al., 2015). However, predicting the evolutionary trajectories of specific glyphosate resistance mutations requires quantifying resistance fitness benefits of particular EPSPS resistance mutations.

In plants, glyphosate targets EPSPS (5‐enolpyruvylshikimate‐3 phosphate synthase) in chloroplasts (Duke & Powles, 2008; Herrmann & Weaver, 1999; Steinrücken & Amrhein, 1980). In evolved glyphosate‐resistant weed ecotypes infesting cropping systems worldwide, specific target site mutations resulting in the substitution of Pro‐106 to 106‐Ala, ‐Leu, ‐Ser, or ‐Thr are well known (Sammons & Gaines, 2014). Recently, a single EPSPS Thr‐102‐Ile substitution has been reported in *Eleusine indica* (Franci et al., 2020) in addition to identification of a novel Thr‐102‐Ser substitution in *Tridax procumbens* (Li et al., 2018).

Glyphosate resistance evolution is complicated by the occurrence of multiple EPSPS resistance mutations, and this makes important the quantification of the relative fitness benefit associated with each of these glyphosate resistance mutations. Distinctive to glyphosate resistance is the possibility of occurrence of multiple EPSPS resistance mutations in a single allele within a single plant (Gaines et al., 2019). Two naturally occurring EPSPS mutations at Thr‐102 and Pro‐106 have been reported. Whereas the first EPSPS variant was identified in *E*.* indica* from Malaysia including the Thr‐102‐Ile +Pro‐106‐Ser (i.e. TIPS) (Yu et al., 2015), the second variant was recently found in *Bidens subalternans* from Paraguay involving the Thr‐102‐Ile +Pro‐106‐Thr (TIPT) (Takano et al., 2020). Interestingly, a rare triple glyphosate resistance EPSPS mutation involving the Thr‐102‐Ile +Ala‐103‐Val +Pro‐106‐Ser codons (TIAVPS) has evolved in *Amaranthus hybridus* from Argentina (Perotti et al., 2019).

Evolution of *E*.* indica* with single (Pro‐106‐Ser) and double (TIPS) glyphosate resistance target site *EPSPS* gene mutations has been recently documented (Yu et al., 2015). The objective of this study is the estimation of resistance fitness benefits associated with these mutational events. Different to previous studies on resistance fitness cost in the absence of glyphosate (Han et al., 2017; Vila‐Aiub et al., 2019), comparison of *E*.* indica* plants sharing a common genetic background and growing in a nonlimiting resource environment under glyphosate selection has enabled, in this current study, an accurate estimation of the glyphosate resistance benefit of EPSPS target site Pro‐106‐Ser vs TIPS mutations and the associated effects on alleles frequencies over time. These results help understand the differential selective advantage and spread rate of these single and double glyphosate resistance *EPSPS* mutations in environments under glyphosate selection.

## 2 MATERIALS AND METHODS

### 2.1 Plant material

*Eleusine indica* is a highly self‐pollinated and genetically diverse diploid (2*n* = 9) annual grass weed with a lack of a specialized dispersal mechanism (Werth et al., 1994). A field evolved glyphosate‐resistant *E*.* indica* population, severely infesting (area >90%) a single palm oil nursery field in Jerantut, Malaysia, was sampled from several patches (Jalaludin et al., 2010, 2014). Sub‐populations used in the present study were previously characterized by Yu et al., (2015) who revealed by sequencing and marker analysis three alleles at the *EPSPS* locus: 102‐Ile/106‐Ser (R), Thr‐102/106‐Ser (r) and the wild type (Thr‐102/Pro‐106) (WT) (Yu et al., 2015). Whereas the R allele exhibits the two EPSPS resistance mutations Thr‐102‐Ile +Pro‐106‐Ser TIPS mutations, the r allele exhibits the single resistance Pro‐106‐Ser mutation (Table 1).

**TABLE 1:** EPSPS mutations, alleles and genotypes identified in the glyphosate‐resistant *Eleusine indica* population used in this study

Mutation	Allele	Genotype^a^	Zygosity
‐	Thr−102/Pro−106 (*wt*)	WT	Homozygous
Pro−106‐Ser	Thr−102/106‐Ser (*r*)	P106S‐rr	Homozygous
Thr−102‐Ile/Pro−106‐Ser	102‐Ile/106‐Ser (*R*)	TIPS‐RR	Homozygous
Thr−102‐Ile/Pro−106‐Ser Pro−106‐Ser	102‐Ile/106‐Ser (*R*) Thr−102/106‐Ser (*r*)	TIPS‐Rr	Compound heterozygous

^a^ Heterozygous genotypes for the *r* or *R* alleles resulting, respectively, in the WT/r and WT/R genotypes were not identified in this population.

Considering the identified three alleles in the collected field population, genetic recombination could potentially result in six genotypes but only four were identified within the single *E*.* indica* population (Table 1) (Yu et al., 2015): glyphosate‐susceptible plants (homozygous WT) and glyphosate‐resistant plants with either the homozygous Pro‐106‐Ser mutation (P106S‐rr) or TIPS (TIPS‐RR) mutation. A fourth genotype was comprised by plants segregating only at the Thr‐102 position but not at the Pro‐106 position rendering plants as compound heterozygous for the TIPS mutation (TIPS‐Rr) (Table 1) (Yu et al., 2015). Neither the genotype heterozygous for the Pro‐106‐Ser mutation (WT/r) nor heterozygous for the TIPS mutation (WT/R) were identified in this population (Yu et al., 2015).

The sub‐populations used in this study arise from the genotyping of plants (*n* = 7–12) which were bulk selfed in isolation in glasshouse conditions to produce seeds which resulted in three purified sub‐populations containing plants with homozygous genotypes of WT, P106S‐rr or TIPS‐RR. Progeny plants (*n* = 10–12) from each of these purified sub‐populations were DNA genotyped using dCAPS markers to confirm their genotype and homozygosity prior to use in the experiments detailed below (Yu et al., 2015). The TIPS‐Rr individuals were identified in seedlings derived from a bulked progeny of Rr × Rr crossing and immediately used in the following experiments described below.

### 2.2 Glyphosate resistance fitness benefit

A number of replicated experiments were carried out to quantify survival, vegetative and reproductive growth of purified TIPS‐RR, TIPS‐Rr, P106S‐rr and WT *E*.* indica* plants under glyphosate selection. Seeds were germinated on 0.7% (w/v) agar at fluctuating temperature 30/20°C with a 12‐h photoperiod and PAR of 200 μmol/m^2^/s. At uniform two‐leaf stage, seedlings were transplanted into pots filled with standard potting mixture (50% composted fine pine bark, 30% coco‐peat and 20% river sand). In all experiments, plants were grown outdoors under average monthly temperatures ranging from 24 to 30°C during the normal growing season (Summer: December–March) of the species.

#### 2.2.1 Plant survival

Twenty‐three seedlings per pot (diameter (*Ø*) = 20 cm, height = 20 cm) at the 4–5 leaf stage, corresponding to purified TIPS‐RR, P106S‐rr and WT, were exposed to increasing glyphosate doses (g/ha): 0 (control), 33.75, 67.5, 135, 270, 540, 1080 (recommended commercial field dose), 2160, 4320, 8640, 17,280, 34,560 and 69,120. Four replicates per dose were used. Overall, the experiment involved 13 glyphosate doses, four replicates and 23 plants per replicate (13 × 4 × 23). Glyphosate (Roundup Attack with IQ inside, 570 g/L, SL; Nufarm Australia) was applied to plants using a cabinet sprayer with a spray volume of 112 L/ha at a pressure of 200 kPa and a speed of 1 m/s. Survival was assessed 4 weeks after glyphosate treatment. Plants were recorded as alive if they were actively growing and tillering after treatment and as dead if there was little new growth and no new tiller formation.

In addition, survival of plants carrying homozygous (RR) vs heterozygous (Rr) TIPS variants in response to the single recommended field glyphosate dose of 1080 g/ha was assessed in three independent experiments. WT and P106S‐rr plants served as controls. Survival data from each experiment were pooled (*n* = 3) accounting for a total of 99, 291, 92 and 92 glyphosate‐treated plants corresponding to WT, P106S‐rr, TIPS‐RR and TIPS‐Rr, respectively. Plants were grown and glyphosate treated as described above. Plant survival was assessed 4 weeks after treatment.

#### 2.2.2 Resource allocation to vegetative growth

An experiment was conducted to quantify the growth of aboveground tissues (stems and leaves) and roots during early growth of individual seedlings in pots (*Ø* = 20 cm, height = 20 cm) treated with 1080 g/ha glyphosate. Ten to fifteen plants per genotype (WT, P106S‐rr, TIPS‐RR) were exposed to glyphosate at the 4–5 leaf stage, and then at weekly intervals, the aboveground foliage and the root biomass were separately harvested from individual plants, washed with tap water, oven‐dried at 60°C for 7 days and weighed. For comparison purposes, growth of WT plants in the absence of glyphosate treatment was also assessed over time. Overall, the experiment involved four harvest times and 10–15 plants per genotype per harvest (4 × 10–15).

Two experiments were conducted to assess total resource partitioning to vegetative biomass assessed in individual plants growing in pots (*Ø* = 30 cm, height = 30 cm) and completing the growth cycle (105 days since germination). Total aboveground vegetative biomass (and seed number, see below) produced by WT, P106‐rr and TIPS‐RR genotypes were evaluated in one experiment, whereas a second experiment quantified the same trait displayed by WT, TIPS‐RR and TIPS‐Rr.

#### 2.2.3 Reproductive growth

Plants (*n* = 5) that survived each of the increasing glyphosate doses (see above) were individually transplanted into pots (*Ø* = 30 cm, height = 30 cm) for quantitative estimation of the number of seeds produced at the end of their annual growth cycle. Inflorescences produced by surviving individuals from each genotype at each glyphosate dose were sequentially collected from first maturity. Inflorescences from each individual were threshed to separate seeds from chaff and rachis material, and total seed mass and number were quantified. Experiments were repeated for further examination of seed production in plants (*n* = 10–20) of all genotypes (P106S‐rr, TIPS‐RR, TIPS‐Rr) exposed to the single recommended glyphosate field dose of 1080 g/ha. The number of seeds (*S_n_*) produced per plant was estimated as:

(1) $$S_{n}=\;\frac{{\mathrm{TS}}_{w}\;100}{S_{w}}$$

where *TS_w_* denotes the total seed weight produced per plant and *S_w_* represents the averaged weight of three aliquots of 100 seeds per individual.

### 2.3 Statistical analysis

Variations in plant survival and associated seed number production over an increasing gradient of glyphosate were analysed by dose–response model with the package drc (Ritz et al., 2015) in R (R‐CoreTeam, 2017). The following three‐parameter log‐logistic function (LL.3 in drc) (Knezevic et al., 2007) was fitted to the data:

(2) $$y=\frac{d}{1+\exp\;(b*(\log(x)‐\log(e))}$$

where *y* denotes plant response (i.e. survival or seed number production) at glyphosate rate *x*, *d* is the upper limit, *b* is the slope at *e* which accounts for the glyphosate dose causing a decrease of 50% survival (LD_50_) or seed number production (*Ysn_50_*) between the upper limit *d* and lower limit. The model fitted with the function drm (dose–response model) (in drc, “dose–response curve” package in R) included glyphosate dose and genotype as independent variables. The glyphosate resistance index (RI) is calculated as the ratio of LD_50_ (or *Ysn_50_*) estimates between the resistant (R) (TIPS‐RR or P106S‐rr) and susceptible (S) (WT) genotypes (IR = LD_50_ (R) / LD_50_ (S)).

Experiments conducted to assess vegetative and reproductive response to the single recommended glyphosate dose (1080 g/ha) were subjected to two‐way analysis of variance (ANOVA) to determine main genotype (WT, P106S‐rr, TIPS‐RR, TIPS‐Rr) and glyphosate (control, 1080 g/ha) effects on traits, using InfoStat statistical software (Di Rienzo et al., 2020). Means were separated using *Tukey's HSD* (honestly significant difference) test (*α* = .05). ANOVA output is provided (Figure S1).

### 2.4 Relative fitness benefit endowed by Pro‐106‐Ser and TIPS mutations

**FIGURE 1:**
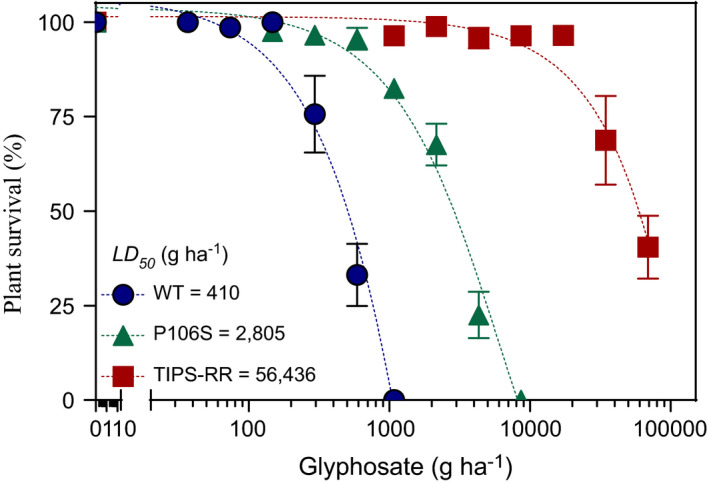
Survival of *Eleusine indica* genotypes carrying the homozygous EPSPS WT (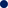), Pro‐106‐Ser (rr) (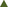) and TIPS‐RR (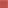) mutation in response to increasing glyphosate doses. Values are mean survival (*n* = 4) and vertical bars denote SE of the mean. Lines represent predicted survival (*S*) derived from logistic model regression analysis: *S*_WT_ = 100/[1 + exp(3.0(log (*x*)‐log (410))]; *S*_P106S_ = 96.7/[1 + exp(2.7(log(*x*)‐log(2806))]; *S*_TIPS_ = 100/[1 + exp(2.1(log (*x*)‐log (56,436))]. Resistance index: P106S = 6.8; TIPS‐RR = 138

**FIGURE 2:**
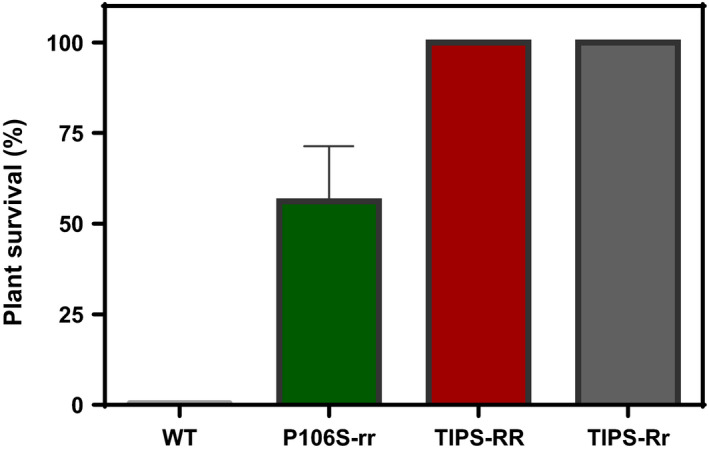
Survival of *Eleusine indica* genotypes carrying the homozygous Pro‐106‐Ser (rr), and homozygous (RR) (102‐Ile +106‐Ser) and heterozygous (Rr) (Thr‐102 / 102‐Ile +106‐Ser) TIPS mutations after treatment with the label dose of 1080 g glyphosate/ha. Values are mean survival pooled from three independent experiments, and vertical bars denote SE of the mean. All experiments were performed under field natural conditions

Fitness (*W*) is a function of both the proportion of plants that survive (*S*) from seed dispersal to reproduction and the amount of offspring or fecundity (*F*) produced by adult plants (*W* = *S* × *F*) (Cousens & Fournier‐Level, 2018; Futuyma, 2013). Fitness of WT, P106‐rr, TIPS‐RR and TIPS‐Rr genotypes was estimated as relative (%) to the fittest genotype:

(3) $$W_{a}=\;\frac{S_{a}F_{a}}{S_{f}.F_{f}}$$

where fitness of genotype *a* (*W_a_*) equals the product of survival (*S_a_*) and fecundity (*F_a_*) of the genotype divided by the product of the survival (*S_f_*) and fecundity (*F_f_*) of the fittest genotype *f*. For the quantitative estimation of relative fitness, survival of WT, P106S‐rr and TIPS‐RR were calculated from the corresponding fitted three‐parameter log‐logistic models in which the glyphosate concentration (×) was fixed at 1080 g/ha (eq. 2) (Figure 1). For the TIPS‐Rr, mean survival was assessed from three pooled experiments in which plants were also exposed to glyphosate at 1080 g/ha under similar environmental conditions (Figure 2). The number of seeds produced by P106S‐rr, TIPS‐RR and TIPS‐Rr was estimated from dedicated experiments conducted with glyphosate at the field dose (Figure 6), except for WT whose fecundity was estimated from the fitted regression model (Figure 5).

### 2.5 Predicted changes in the frequency of EPSPS alleles under glyphosate selection

Changes in the frequency of *wt*, *r* and *R* alleles were modelled assuming an environment under recurrent glyphosate selection with the field dose (1080 g/ha) over *E*.* indica* generations. *Eleusine indica* (2*n*= 2*x* =18) is an annual species and the prediction model assumed a single glyphosate treatment per generation or calendar year. Initial allelic frequencies were elaborated to resemble a glyphosate‐susceptible population at the onset of the glyphosate selective process and therefore assumed as 0.999, 1 × 10^−6^ and 1 × 10^−12^ for the *wt*, *r* (single mutation) and *R* (double mutation) alleles, respectively. The assumed initial frequency of the nuclear‐encoded EPSPS r and R alleles are associated with theoretical and empirical estimations of herbicide resistance mutation frequencies (Casale et al., 2019; Diggle & Neve, 2001; Gressel & Segel, 1990; Jasieniuk et al., 1996).

The combination of the EPSPS *wt*, *r* and *R* alleles found in the field‐collected *E*.* indica* population define six genotypes (Figure S2). As the heterozygous WT/r and WT/R genotypes were not detected in the field population, the matrix of relative fitness was completed assuming that the heterozygous genotypes for the glyphosate resistance *r* and *R* alleles exhibit incomplete dominance (0.5) compared with the estimated relative fitness of the homozygous WT and TIPS‐RR genotypes (Huffman et al., 2016) (Figure S2).

The expected frequency of *wt*, *r* and *R* alleles in the next generation after glyphosate selection are REF(Allendorf et al., 2009):

(4) $$wt=\frac{\left(f_{{\left(wt\right)}^{2}}\;w_{\left(WT\right)}+f_{\left(wt*r\right)}w_{\left(WT/r\right)}+f_{\left(wt*R\right)}w_{\left(WT/R\right)}\right)}{\bar{w}}$$

(5) $$r=\frac{\left(f_{\left(wt*r\right)}\;w_{\left(WT/r\right)}+f_{{\left(r\right)}^{2}}w_{\left(P106S‐rr\right)}+f_{\left(r*R\right)}w_{\left(TIPS‐Rr\right)}\right)}{\bar{w}}$$

(6) $$R=\frac{\left(f_{\left(wt*R\right)}\;w_{\left(WT/R\right)}+f_{\left(r*R\right)}w_{\left(TIPS‐Rr\right)}+f_{{\left(R\right)}^{2}}w_{\left(TIPS‐RR\right)}\right)}{\bar{w}}$$

where *f* denotes de frequency of alleles *wt*, *r* and *R*, *w* is the relative fitness of the three homozygous WT, P106S‐rr and TIPS‐RR and three heterozygous WT/r, WT/R and TIPS‐Rr genotypes and $\bar{w}$ is the average fitness of the *E*.* indica* population. Calculations were conducted assuming no overlapping generations in a large population, and absence of migration or back mutation.

The average fitness ($\bar{w}$) of the *E*.* indica* population is calculated as the sum of the products of the frequency of each genotype multiplied by its relative fitness frequency (Allendorf et al., 2009):

(7) $$\begin{array}{c} \bar{w}=f_{\left(WT\right)}w_{\left(WT\right)}+f_{\left(WT/r\right)}w_{\left(WT/r\right)}+f_{\left(P106S‐rr\right)}\;w_{\left(P106S‐rr\right)}+\\ f_{\left(WT/R\right)}w_{\left(WT/R\right)}+f_{\left(TIPS‐Rr\right)}w_{\left(TIPS‐Rr\right)}+f_{\left(TIPS‐RR\right)}w_{\left(TIPS‐RR\right)} \end{array}$$

When the estimated frequencies of EPSPS *wt*, *r* and *R* allelic frequencies showed no further generational changes and reached equilibrium, the frequency of EPSPS genotypes were estimated using a model that accounts for a departure from Hardy–Weinberg due to nonrandom mating condition to reflect the *E*.* indica* inbreeding system (Futuyma, 2013). As the *wt* allele was predicted to be lost in the population after glyphosate selection (see Results), the genotypic frequencies were estimated using an EPSPS locus with the r and R alleles:

$$P106S‐\mathrm{rr}=r^{2}+FrR$$

$$\mathrm{TIPS}‐\mathrm{RR}=R^{2}\;+FrR$$

$$\mathrm{TIPS}‐\mathrm{Rr}=2rR\;\left(1,‐,F\right)$$

where P106S‐rr, TIPS‐RR and TIPS‐Rr denote the EPSPS genotypes, *r* and *R* are the predicted frequency of EPSPS glyphosate resistance alleles after glyphosate selection and *F* is the inbreeding coefficient. A high inbreeding coefficient (*F*) was assumed (*F* = 0.97) to reflect the species reproductive biology.

## 3 RESULTS

### 3.1 Plant survival

The logistic model provided a significant data fit (*p* < 0.0001) associated with variations in survival to increasing glyphosate doses for all genotypes. As expected, glyphosate dose treatments revealed differential survival of P106S‐rr versus TIPS‐RR genotypes (Figure 1). At field (1080 g glyphosate/ha) or extremely high (10,000 g glyphosate/ha) doses no WT and P106S‐rr plants survived. However, the TIPS‐RR plants exhibited a remarkable ability to survive glyphosate at doses even higher than 20,000 g/ha. Thus, the LD_50_ (g/ha) for WT, P106S‐rr and TIPS‐RR genotypes were 410, 2800 and 56,436 g/ha, respectively (Figure 1). The estimated resistance index for the P106S‐rr and TIPS‐RR was 6.8 ± 0.6 and 138 ± 13, respectively. At the recommended glyphosate field dose (1080 g/ha), both heterozygous (Rr) and homozygous (RR) TIPS plants showed 100% survival, whereas survival of plants with the P106S‐rr genotype was 56% (Figure 2).

### 3.2 Resource allocation to vegetative growth

**FIGURE 3:**
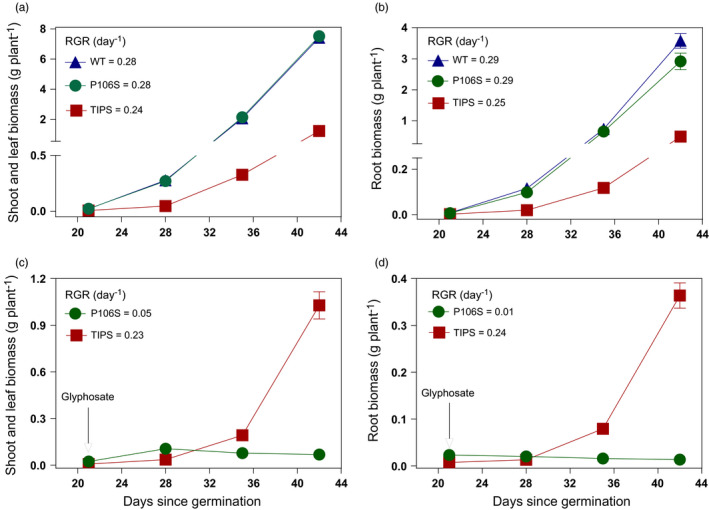
Aboveground and root biomass produced overtime under field conditions by EPSPS WT (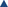), homozygous P106S‐rr (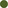) and homozygous TIPS‐RR 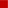) genotypes in the absence (a‐b) and presence (c‐d) of glyphosate treatment (1080 g/ha). Data are mean (*n* = 10–15) with standard errors as vertical bars. Relative growth rate (RGR) was estimated over a 3‐week growth period. Arrows denote the time of glyphosate treatment

**FIGURE 4:**
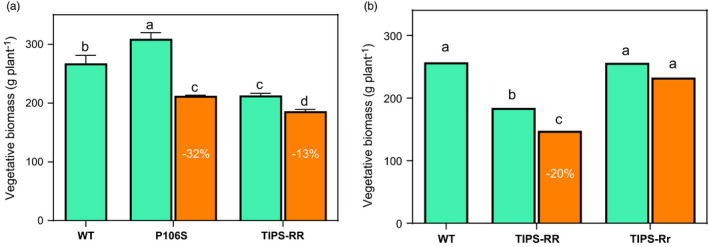
Aboveground vegetative (leaf +stem) biomass produced by P106S, TIPS‐RR and TIPS‐Rr genotypes at plant maturity (105 days of growth since seed germination). Orange bars(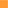) are mean estimates under glyphosate treatment (1080 g/ha). Percentage values (%) denote significant biomass reductions relative to the biomass produced under no glyphosate treatment (green bars 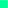). For comparison purposes, aboveground vegetative biomass produced by WT plants under no glyphosate treatment (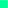) was also assessed. Letters A and B correspond to independent experiments conducted in the same summer growing season. Vertical bars denote SE of the mean (*n* = 10–20). Different letters indicate significant differences among genotypes according to Tukey's HSD test (*α* = 5%)

Vegetative growth of glyphosate‐surviving plants (1080 g/ha) showed significant differences between the genotypes. Surviving P106S‐rr plants exhibited a major reduction in shoot/leaves (Figure 3c) and root (Figure 3d) growth following glyphosate treatment compared with surviving TIPS‐RR (Figure 3c,d). The impaired vegetative growth associated with the P106S‐rr genotype was correlated with a lower relative growth rate (RGR) of shoots and roots over 3 weeks after glyphosate treatment which accounted for about 99% aerial and root biomass reduction compared with glyphosate untreated P106S‐rr plants (Figure 3a,b). At plant maturity (i.e. 15 weeks after seed germination), P106S‐rr plants showed some growth recovery from glyphosate, displaying a 32% reduction in total vegetative biomass compared with untreated plants (Figure 4a).

TIPS‐RR plants showed a marginal decrease in RGR displaying about 15% reduction in both the aboveground and root biomass compared with glyphosate untreated TIPS‐RR plants 3 weeks after glyphosate treatment (Figure 3c,d vs 3a,b). At maturity, TIPS‐RR showed a reduction in vegetative biomass that ranged from 20% to 13% compared with glyphosate untreated plants (Figure 4a,b).

Unlike homozygous TIPS‐RR, heterozygous TIPS‐Rr plants treated with the glyphosate field dose (1080 g/ha) displayed no apparent reduction in vegetative growth as evidenced by the similar total biomass produced compared with untreated plants at maturity (Figure 4b).

It is noteworthy highlighting the notable growth reduction associated with TIPS‐RR but not with TIPS‐Rr when compared to WT in the absence of glyphosate treatment (Figures 3a,b and 4a,b), denoting an adaptive fitness cost associated with the homozygous TIPS mutation.

### 3.3 Resource allocation to reproduction

**FIGURE 5:**
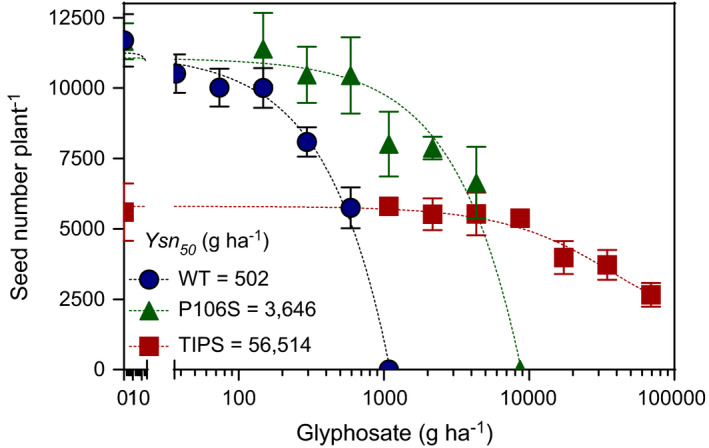
Variation in the number of seeds produced by *Eleusine indica* genotypes carrying the homozygous EPSPS WT(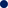), Pro‐106‐Ser (rr) (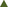) and TIPS‐RR (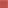) mutation over increasing glyphosate doses. Values are mean (*n* = 5) seed number estimates, and vertical bars denote SE of the mean. Lines represent predicted survival (*S*) derived from logistic model regression analysis: *S*WT = 10,575/[1 + exp(2.65(log (*x*)−log (502))]; SP106S = 10,970/[1 + exp(1.66(log (*x*)‐log (3646))]; *S*TIPS = 5781/[1 + exp(1.03(log (*x*)−log (56,514))]. Resistance index: P106S = 7.3; TIPS‐RR = 113

Increases in glyphosate dose reduced the number of seeds produced by individuals of WT, P106S‐rr and TIPS‐RR genotypes (Figure 5). The reduction in seed number over increasing glyphosate doses followed a similar pattern to survival exhibited by WT and P106S‐rr resulting in a *Ysn_50_* of 502 and 3646 g/ha of glyphosate (Figure 5). The seed production showed by TIPS‐RR plants was notably lower than WT and P106S‐rr in the absence of glyphosate treatment and remained unaffected until glyphosate doses increased above 8500 g/ha (Figure 5). Thus, the *Ysn_50_* for TIPS‐RR was 56,514 g/ha. The estimated resistance index based on seed production as compared to WT for the P106S‐rr and TIPS‐RR was P106S = 7.3 ± 1.5 and 113 ± 53, respectively.

**FIGURE 6:**
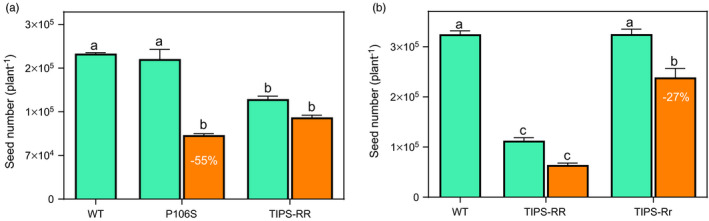
Number of seeds produced by P106S, TIPS‐RR and TIPS‐Rr genotypes at the end of growth cycle (105 days of growth since seed germination). Orange bars (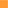) are mean estimates under glyphosate treatment (1080 g/ha). Percentage values (%) denote significant seed number reductions relative to the number of seeds produced under no glyphosate treatment (green bars 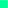). For comparison purposes, the number of seeds produced by WT plants under no glyphosate treatment 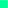) was also assessed. a and b are independent experiments conducted in the same summer growing season. Vertical bars denote SE of the mean (*n* = 10–20). Different letters indicate significant differences among genotypes according to Tukey’s HSD test (*α* = 5%)

A detrimental effect of glyphosate on reducing the reproductive potential of P106S‐rr genotype was observed. P106S‐rr plants showed a 55% reduction in the ability to produce seeds when exposed to the glyphosate field dose ($\bar{x}$ = 100,000 seeds) compared with untreated plants ($\bar{x}$ = 220,000 seeds) (Figure 6a). In contrast, TIPS‐RR plants showed no significant reduction in the number of seeds produced when exposed to the field rate of glyphosate ($\bar{x}$ = 130,000 seeds) compared with untreated plants ($\bar{x}$ = 160,000 seeds). Conversely, seed number in TIPS‐RR was notably lower than WT and P106S‐rr in glyphosate free environment (Figure 6a).

Although showing a 27% reduction in the number of seeds compared with the untreated condition, heterozygous TIPS‐Rr plants still exhibited a greater number of seeds than homozygous TIPS‐RR when treated with the glyphosate field dose (1080 g/ha) (Figure 6b). Under no glyphosate treatment, heterozygous TIPS‐Rr plants showed similar and higher seed production compared with WT and homozygous TIPS‐RR, respectively (Figure 6b).

### 3.4 Relative fitness benefit endowed by Pro‐106‐Ser and TIPS mutations

Plant survival and fecundity mean estimates of P106S‐rr and TIPS‐Rr and TIPS‐RR genotypes under glyphosate selection (1080 g/ha) enabled the quantification of overall glyphosate resistance benefit endowed by each of the *EPSPS* gene variants in *E*.* indica* (Table 2). The highest resistance benefit was associated with the heterozygous TIPS‐Rr genotype which showed no mortality and a mean estimate of 237,000 seeds under glyphosate treatment. Plants of the homozygous P106S‐rr and TIPS‐RR genotypes exhibited, respectively, only 31% and 39% of the resistance benefit exhibited by the TIPS‐Rr plants (Table 2).

**TABLE 2:** Estimates of glyphosate resistance benefits associated with *Eleusine indica* WT, P106S, homozygous RR or heterozygous Rr TIPS genotypes

Genotype	Survival (*S*)	Fecundity^c^ (*F*)	Relative fitness (*W*)^d^
WT	0.21^a^	3096^a^	0.002
P106S‐rr	0.73^a^	101,130^b^	0.31
TIPS‐RR	0.97^a^	96,000^b^	0.39
TIPS‐Rr	1.0^b^	237,000^b^	1.0

Note

Relative fitness (W) of *W*_WT_ = (*S*_WT_ × *F*_WT_ / *S*_TIPS‐Rr_ × *F*_TIPS‐Rr_).

Relative fitness (*W*) of *W*_TIPS‐RR_ = (*S*_TIPS‐RR_ × *F*_TIPS‐RR_ / *S*_TIPS‐Rr_ × *F*_TIPS‐Rr_).

Relative fitness (*W*) of *WP_106S_*_‐rr_ = *S_106S_*_‐rr_ × *F_106S_*_‐rr_ / *S*_TIPS‐Rr_ × *F*_TIPS‐Rr_).

Abbreviations: *F*, fecundity; *S*, survival.

^a^ Survival and fecundity parameters calculated from the log‐logistic 3‐parameter regression equations at the recommended glyphosate dose of 1080 g/ha.

^b^ Survival and fecundity parameters calculated from the mean estimate of three independent experiments with plants treated at the recommended glyphosate dose of 1080 g/ha.

^c^ Fecundity is the average number of seeds produced per single plants.

^d^ Fitness (*W*) was estimated as relative to the fittest genotype (TIPS‐Rr) and normalized to *W*_TIPS‐Rr_ = 1.

### 3.5 Predicted changes in the frequency of EPSPS alleles under glyphosate selection

**FIGURE 7:**
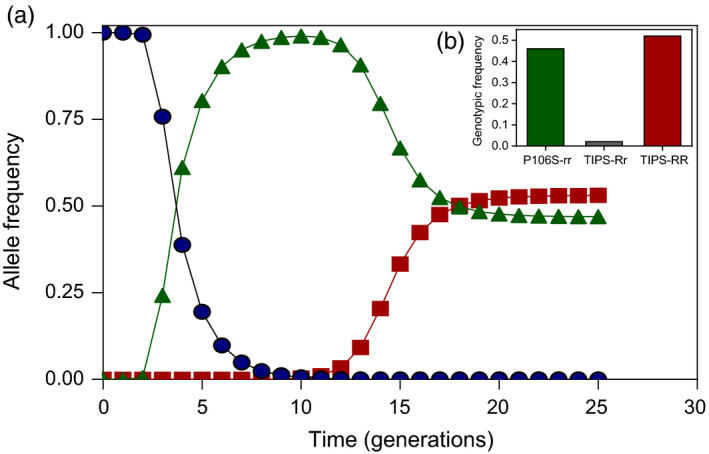
Predicted changes in the frequency of *EPSPS wt* (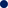), r (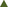) and R (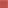) alleles under continuous glyphosate selection (1080 g/ha) over time (a). Predicted genotypic frequencies at equilibrium reached after glyphosate selection (b)

The frequency of the *wt* allele started to decline after three generations under glyphosate use coinciding with a steep increase in the frequency of the *r* allele. After 10 generations under continuous glyphosate selection, the estimated *wt* and glyphosate resistance *r* allele frequencies showed, respectively, nearly complete loss (*wt* =0.006) and fixation (*r* = 0.99) in the population (Figure 7). The frequency of the resistance TIPS *R* allele showed increases that were only noticeable from the 12th generation. From generation 12, the frequency of both glyphosate resistance *r* and TIPS *R* alleles started to decline and increase, respectively, reaching a stable equilibrium with no further allelic changes at 24th generation with frequencies of 0.47 (*r* allele) and 0.53 (*R* allele) (Figure 7).

After 24 years of glyphosate selection, the predicted frequencies of glyphosate‐resistant genotypes were 0.46, 0.02 and 0.52 for the homozygous P106S‐rr, compound heterozygous TIPS‐Rr and homozygous TIPS‐RR, respectively (Figure 7). Variations in the inbreeding coefficient in the range of 0.90 to 0.99 had little effect on estimates of the genotypic frequencies at equilibrium after glyphosate selection (data not shown).

## 4 DISCUSSION

The results of this study indicate that the glyphosate resistance single (P106S) and double (TIPS) target site EPSPS mutations confer contrasting plant fitness benefits in *E*.* indica* plants under glyphosate selection. At recommended glyphosate field dose, (1) P106S‐rr plants show reduced survival and compromised growth and fecundity compared with TIPS plants and (2) whereas both homozygous TIPS‐RR and heterozygous TIPS‐Rr plants displayed similar glyphosate survival rates, a significantly higher fecundity is present in TIPS‐Rr than TIPS‐RR plants. In addition, (3) in a glyphosate‐free environment, only plants with the TIPS‐RR genotype exhibit a severe growth and reproduction limitation when compared to P106S‐rr and TIPS‐Rr plants and WT, denoting a significant recessive fitness cost associated with the homozygous TIPS mutation (Han et al., 2017).

### 4.1 A higher glyphosate resistance benefit is associated with the TIPS than P106S mutation

Recent published studies have revealed that, at the enzyme level, the *Escherichia coli* expressed *E*.* indica* homozygous Pro‐106‐Ser and Thr‐102‐Ile/Pro‐106‐Ser TIPS mutations differ in their susceptibility to glyphosate inhibition (Yu et al., 2015). Whereas the glyphosate inhibitory concentration (IC_50_) of Pro‐106‐Ser was 87 μM, the IC_50_ value was *c*. 53,000 μM for the TIPS variant (Yu et al., 2015). Similarly, various studies determining both the IC_50_ and inhibition constant (*K*_i_) demonstrate that the resistance EPSPS maize Pro‐106‐Ser variant is more glyphosate susceptible than the TIPS variant (Alibhai et al., 2010; T Funke et al., 2009; Robert Douglas Sammons & Gaines, 2014). Molecular modelling by Funke et al., (2009) revealed that TIPS is more efficient than Pro‐106‐Ser in reducing glyphosate binding to EPSPS. These results provide the fundamental biochemical and molecular bases for the observed differential glyphosate resistance benefit at the plant level in the P106S‐rr and TIPS‐RR EPSPS variants.

At the plant level, the highest plant resistance fitness benefit is expected when a herbicide resistance trait minimizes herbicide binding to a target enzyme without any adverse effects to enzyme efficacy. A number of experiments conducted here have shown that P106S‐rr or TIPS plants differ in their overall plant fitness when exposed to commercial field glyphosate dose of 1080 g/ha. P106S‐rr plants not only showed about 30% mortality following glyphosate treatment, but also those surviving individuals exhibited a significant reduction in vegetative and reproductive growth compared with untreated plants. Conversely, homozygous TIPS‐RR genotype showed zero mortality when exposed to 1080 g/ha of glyphosate and only a limited reduction in the vegetative growth with no significant impact on reproductive fitness compared with glyphosate‐untreated plants.

However, an even higher glyphosate resistance fitness benefit is associated with the compound heterozygous TIPS‐Rr compared with TIPS‐RR plants. Although both Rr and RR TIPS plants show no plant mortality at 1080 g/ha glyphosate, vegetative and reproductive growth has been shown to be significantly higher in TIPS‐Rr than in TIPS‐RR plants. The molecular basis for the advantageous interaction between the EPSPS R and r alleles yielding the highest glyphosate resistance fitness benefit at the plant level in TIPS‐Rr is unknown. The TIPS‐Rr genotype encompasses the best protection to glyphosate damage and expresses no fitness costs in environments under no glyphosate treatment (Han et al., 2017).

These results enable us to rank the three EPSPS genotypes for their overall contribution to plant fitness assessed under glyphosate selection: TIPS‐Rr >> TIPS‐RR >P106S‐rr (Table 2). Further research is required to determine the EPSPS glyphosate sensitivity (Ki, IC_50_) of the TIPS‐Rr genotype and thus correlate the resistance benefit at both the enzyme and plant levels as has been shown for the TIPS‐RR and Pro‐106‐Ser mutations.

These results comprise the first experimental evidence of a resistance benefit conferred by different EPSPS target site glyphosate resistance mutations in naturally evolved glyphosate‐resistant weeds in agroecosystems.

### 4.2 Evolutionary and ecological significance of results

Understanding the evolutionary ecology of target site mutations endowing glyphosate resistance in agricultural weeds is fundamental to predict the trajectory of resistance evolution. The likelihood of spread and fixation of novel herbicide resistance mutations in agroecosystems depends on their impact on plant survival and fecundity (i.e. fitness) under both the presence (resistance benefit) or absence (resistance cost) of herbicide selection (see reviews by Bergelson & Purrington, 1996; Jasieniuk et al., 1996; Vila‐Aiub et al., 2009, 2011). It is clear that the value of herbicide resistance benefit plays an evolutionary role in favouring the rapid genetic fixation of the fittest resistance alleles. Hence, differences in resistance benefits among resistance traits define the rate of enrichment and equilibrium frequency in areas under herbicide selection.

In an environment under persistent glyphosate selection, our estimations predict that the EPSPS resistance *r* (Pro‐106‐Ser) and *R* (TIPS) glyphosate resistance‐endowing alleles will increase their frequency, as expected, at the expense of the glyphosate susceptible *wt* allele which is nearly lost after 10 years of selection. However, despite the associated lower resistance benefit, the rate of enrichment of the *r* allele will be higher than the TIPS *R* allele for the first 10 years of glyphosate selection, driven by the likely higher initial frequency (1 × 10^−6^) before selection compared with the rarer frequency of the TIPS *R* allele (1 × 10^−12^). The TIPS *R* allele is predicted to reach the highest frequency of 0.53 after 20 years of glyphosate selection in stable equilibrium with the resistance *r* allele.

The studied *E*.* indica* population collected from the field has been shown to comprise 49% of plants with the heterozygous TIPS‐Rr genotype, 34% P106S‐rr, 16% WT and 1.6% homozygous TIPS‐RR (Yu et al., 2015). However, our modelling exercise predicts that, in a highly inbreeding species like *E*.* indica* and after 20 years of glyphosate selection, only 2% of individuals would display the heterozygous TIPS‐Rr genotype, despite the highest associated fitness benefit. In contrast, homozygous plants with the TIPS‐RR and P106S‐rr genotypes would comprise 52% and 46% of the population, respectively.

The discrepancies between observed and predicted EPSPS genotypic frequencies may represent unstable transient frequencies observed in the field that may lead to the loss of WT, and a small fraction of TIPS‐Rr at the expense of TIPS‐RR and P106S‐rr over generations. Alternatively, the higher frequency of plants segregating at the Thr‐102 position (TIPS‐Rr) observed in the field may be the result of sequential mutational events occurring at higher rates than expected where the Thr‐102‐Ile mutation integrates in plants already harbouring the homozygous P106S‐rr mutation (Sammons & Gaines, 2014; Yu et al., 2015).

If other alternative weed control tools are not implemented (Vila‐Aiub, 2019), given the differences in survival, growth and reproductive fitness between TIPS‐Rr, TIPS‐RR and P106S‐rr, and considering equally similar starting low frequencies in the field, any attempt to control *E*.* indica* populations harbouring these genotypes with increasing glyphosate doses would select and enrich faster for the TIPS‐Rr followed by TIPS‐RR and then P106S‐rr genotypes. Although the high inbreeding observed in *E*.* indica* would dilute the presence of the fittest heterozygous TIPS‐Rr in favour of the homozygous TIPS‐RR and P106S‐rr.

## CONFLICT OF INTEREST

The authors declare no conflict of interest.

## Supporting information

Fig S1

Fig S2

## Data Availability

The data that support the findings of this study are available from the corresponding author upon reasonable request.
