# Supplementary material for: Contrasting plant ecological benefits endowed by naturally occurring EPSPS resistance mutations under glyphosate selection
Source: Evol Appl. 2021 Mar 29;14(6):1635–45. doi: 10.1111/eva.13230 (PMC8210788; doi:10.1111/eva.13230)
Supplement: Supplementary file 1 — Fig S1 [file EVA-14-1635-s002.docx]

**Analysis of variance (WT vs P106S vs TIPS-RR)**

Variable N R² Adj R² CV

**biomass** 60 0.81 0.80 8.93

**Analysis of variance table (Sequential SS)**

S.V. SS df MS F p-value

Model 101543.08 4 25385.77 58.57 <0.0001

Gly 48719.61 1 48719.61 112.40 <0.0001

Gen 40425.41 2 20212.71 46.63 <0.0001

Gly*Gen 12398.05 1 12398.05 28.60 <0.0001

Error 23840.03 55 433.46

Total 125383.10 59

**Test:Tukey Alpha:=0.05 LSD:=10.77293**

*Error: 433.4550 df: 55*

Gly Means n S.E.

1080 199.63 30 4.03 A

0 261.69 30 3.80 B

*Means with a common letter are not significantly different (p > 0.05)*

**Test:Tukey Alpha:=0.05 LSD:=17.53231**

*Error: 433.4550 df: 55*

Gen Means n S.E.

TIPS 197.91 20 4.66 A

106 261.28 30 4.03 B

WT 265.96 10 6.58 B

*Means with a common letter are not significantly different (p > 0.05)*

**Test:Tukey Alpha:=0.05 LSD:=24.91196**

*Error: 433.4550 df: 55*

Gly Gen Means n S.E.

1080 WT nd 0 nd A

1080 TIPS 184.41 10 6.58 B

0 TIPS 211.40 10 6.58 C

1080 106 214.84 20 4.66 C

0 WT 265.96 10 6.58 D

0 106 307.71 10 6.58 E

*Means with a common letter are not significantly different (p > 0.05)*

**Analysis of variance (WT vs P106S vs TIPS-RR)**

Variable N R² Adj R² CV

**seed** 35 0.73 0.70 21.35

**Analysis of variance table (Sequential SS)**

S.V. SS df MS F p-value

Model 88237417143.56 4 22059354285.89 20.73 <0.0001

Gly 70691359113.64 1 70691359113.64 66.43 <0.0001

Gen 6622232336.75 2 3311116168.37 3.11 0.0592

Gly*Gen 10923825693.16 1 10923825693.16 10.26 0.0032

Error 31925727757.81 30 1064190925.26

Total 120163144901.37 34

**Test:Tukey Alpha:=0.05 LSD:=22756.04849**

*Error: 1064190925.2604 df: 30*

Gly Means n S.E.

1080 119134.88 20 8422.95 A

0 204692.90 15 8422.95 B

*Means with a common letter are not significantly different (p > 0.05)*

**Test:Tukey Alpha:=0.05 LSD:=38847.48277**

*Error: 1064190925.2604 df: 30*

Gen Means n S.E.

TIPS 144293.24 10 10315.96 A

106 165935.09 20 8422.95 A

WT 231891.79 5 14588.97 B

*Means with a common letter are not significantly different (p > 0.05)*

**Test:Tukey Alpha:=0.05 LSD:=55712.78159**

*Error: 1064190925.2604 df: 30*

Gly Gen Means n S.E.

1080 WT nd 0 nd A

1080 106 108621.34 15 8422.95 B

1080 TIPS 129648.43 5 14588.97 B

0 TIPS 158938.06 5 14588.97 B

0 106 223248.84 5 14588.97 C

0 WT 231891.79 5 14588.97 C

*Means with a common letter are not significantly different (p > 0.05)*

**Analysis of variance (TIPS-RR vs TIPS-Rr)**

Variable N R² Adj R² CV

**Biomass** 48 0.73 0.70 12.55

**Analysis of variance table (Sequential SS)**

S.V. SS df MS F p-value

Model 80048.41 4 20012.10 28.86 <0.0001

Gly 15472.23 1 15472.23 22.31 <0.0001

Gen 64143.91 2 32071.95 46.25 <0.0001

Gly*Gen 432.26 1 432.26 0.62 0.4341

Error 29819.89 43 693.49

Total 109868.30 47

**Test:Tukey Alpha:=0.05 LSD:=15.54838**

*Error: 693.4858 df: 43*

Gly Means n S.E.

1080 188.53 20 5.89 A

0 226.90 28 5.00 B

*Means with a common letter are not significantly different (p > 0.05)*

**Test:Tukey Alpha:=0.05 LSD:=23.66390**

*Error: 693.4858 df: 43*

Gen Means n S.E.

TIPS 164.30 20 5.89 A

Heterozygous 242.65 18 6.25 B

WT 243.87 10 8.33 B

*Means with a common letter are not significantly different (p > 0.05)*

**Test:Tukey Alpha:=0.05 LSD:=34.35568**

*Error: 693.4858 df: 43*

Gly Gen Means n S.E.

1080 WT nd 0 nd A

1080 TIPS 145.97 10 8.33 B

0 TIPS 182.63 10 8.33 C

1080 Heterozygous 231.10 10 8.33 D

0 WT 243.87 10 8.33 D

0 Heterozygous 254.21 8 9.31 D

*Means with a common letter are not significantly different (p > 0.05)*

**Analysis of variance**

Variable N R² Adj R² CV

**Seeds** 25 0.96 0.95 12.19

**Analysis of variance table (Sequential SS)**

S.V. SS df MS F p-value

Model 289820945335.86 4 72455236333.96 109.16 <0.0001

Gly 63248663159.08 1 63248663159.08 95.29 <0.0001

Gen 224798281687.01 2 112399140843.50 169.34 <0.0001

Gly*Gen 1774000489.77 1 1774000489.77 2.67 0.1177

Error 13275121625.87 20 663756081.29

Total 303096066961.72 24

**Test:Tukey Alpha:=0.05 LSD:=21939.93329**

*Error: 663756081.2933 df: 20*

Gly Means n S.E.

1080 149726.58 10 8147.12 A

0 252398.12 15 6652.10 B

*Means with a common letter are not significantly different (p > 0.05)*

**Test:Tukey Alpha:=0.05 LSD:=33659.37291**

*Error: 663756081.2933 df: 20*

Gen Means n S.E.

TIPS 86611.66 10 8147.12 A

Heterozygous 280168.77 10 8147.12 B

WT 323086.65 5 11521.77 C

*Means with a common letter are not significantly different (p > 0.05)*

**Test:Tukey Alpha:=0.05 LSD:=48758.48531**

*Error: 663756081.2933 df: 20*

Gly Gen Means n S.E.

1080 WT nd 0 nd A

1080 TIPS 62366.10 5 11521.77 B

0 TIPS 110857.23 5 11521.77 B

1080 Heterozygous 237087.07 5 11521.77 C

0 WT 323086.65 5 11521.77 D

0 Heterozygous 323250.47 5 11521.77 D

*Means with a common letter are not significantly different (p > 0.05)*
