# Supplementary material for: Contrasting plant ecological benefits endowed by naturally occurring EPSPS resistance mutations under glyphosate selection
Source: Evol Appl. 2021 Mar 29;14(6):1635–45. doi: 10.1111/eva.13230 (PMC8210788; doi:10.1111/eva.13230)
Supplement: Supplementary file 2 — Fig S2 [file EVA-14-1635-s001.docx]

***Supplementary Figure 2***

A. EPSPS (*wt*, *r*, *R*)^#^ alleles and definition of the six *Eleusine indica* genotypes

| **Genotypes** | |  | Alleles^#^ |  |
| --- | --- | --- | --- | --- |
|  |  | ***wt*** | ***r*** | ***R*** |
|  | ***wt*** | WT | WT/r | WT/R |
| Alleles^#^ | ***r*** | r/WT | rr | **rR** |
|  | ***R*** | R/WT | **Rr** | RR |

| ^#^Allele *wt* correspond to wild type  ^#^Allele *r* corresponds to the single mutation Pro-106-Ser  ^#^Allele *R* denotes the double TIPS mutation Thr-102-Ile + Pro-106-Ser |
| --- |

B. Defined genotypes and corresponding names

C. Matrix of genotypic relative fitness

| **Genotypic fitness matrix** | |  | Alleles |  |
| --- | --- | --- | --- | --- |
|  |  | ***wt*** | ***r*** | ***R*** |
|  | ***wt*** | 0.002* | 0.155^ | 0.195^ |
| Alleles | ***r*** | 0.155^ | 0.31* | **1.0*** |
|  | ***R*** | 0.195^ | **1.0*** | 0.39* |

*Relative fitness estimates associated with WT, P106S-rr, TIPS-RR and TIPS-Rr from Table 2

^Estimates of relative fitness associated with heterozygous WT/r and WT/R are assumed to have a semi-dominant fitness (0.5) in relation to parental homozygous resistant (P106S-rr or TIPS-RR) and susceptible (WT) genotypes
